# Development of the National Policy for Quality in Healthcare for Malaysia

**DOI:** 10.1186/s12961-023-01063-w

**Published:** 2023-11-14

**Authors:** Samsiah Awang, Bruce Agins, Izzatur Rahmi Mohd Ujang, Divya Nair Narayanan, Nur Wahida Zulkifli, Normaizira Hamidi

**Affiliations:** 1 Centre for Healthcare Quality Research, Institute for Health Systems Research, National Institutes of Health, Ministry of Health Malaysia, Block B2, NIH Complex, No. 1, Jalan Setia Murni U13/52, Setia Alam, 40170 Shah Alam, Selangor Malaysia; 2 Institute for Global Health Sciences, Division of Epidemiology, University of California San Francisco, 550 16th Street, San Francisco, CA 94158 USA; 3 Faculty of Pharmacy, Universiti Teknologi MARA, Puncak Alam Campus, FF1, Level 11, Bandar Puncak Alam, 42300 Puncak Alam Selangor Malaysia

**Keywords:** Policy, Strategy, Quality, Healthcare, Malaysia

## Abstract

**Background:**

Quality in healthcare is a fundamental pillar of health systems performance, leading to improved health outcomes and reduced waste. The World Health Organization recommends that countries establish a national quality policy and strategy (NQPS) to steer the provision of safe and high-performing healthcare services and foster a quality culture. This paper describes the development process and key content of Malaysia’s new 5-year National Policy for Quality in Healthcare.

**Methods:**

The development process was managed by a technical working group led by the Institute for Health Systems Research in the Ministry of Health. Situational analysis was conducted through a multi-pronged approach, underpinned by a review of the past and present healthcare sectoral and quality plans and guided by the WHO NQPS framework. This approach involved: (i) review of quality-related policy documents, (ii) online surveys of healthcare providers and the public, (iii) key-informant facilitated discussions and (iv) mapping of existing quality improvement initiatives (QIIs). Data gathered from these approaches informed the content of the new policy. Following thematic analysis, the findings were grouped into specific domains, which were then organized into a strengths, weaknesses, opportunities, and threats (SWOT) framework.

**Results:**

Ten key areas of concern identified were (i) a people-centred holistic approach, (ii) governance for quality, (iii) resources, (iv) quality culture, (v) stakeholder engagement, (vi) health management information system, (vii) workforce competency, (viii) knowledge exchange, (ix) quality indicators and (x) monitoring and evaluation of quality activities. These led to the formulation of seven strategic priorities  for the planning of improvements aimed at addressing the key areas of concern. The national definition of quality was affirmed. A total of 40 QIIs were mapped and grouped into three broad categories, namely (i) regulatory, (ii) domain-specific QIIs and (iii) Quality Improvement (QI) method.

**Conclusions:**

The National Policy for Quality in Healthcare for Malaysia was developed through a comprehensive situational analysis using a multi-method approach that identified priorities across national, state, institutional and community levels. This evidence-informed approach led to meaningful contextual adaptation of the NQPS framework to shape the strategic direction to advance quality and achieve effective and safe outcomes for all Malaysians.

**Supplementary Information:**

The online version contains supplementary material available at 10.1186/s12961-023-01063-w.

## Background

High-quality care improves health outcomes and reduces waste. Conversely, poor-quality care is responsible for most deaths caused by health-related conditions, surpassing the proportion attributable to poor health system access [1]. Quality becomes a fundamental component of all global health activities to achieve the Sustainable Development Goals through Universal Health Coverage (UHC). Developing a national plan and strategy for healthcare quality offers an important vehicle for reaching these goals.

Achievement of health outcomes throughout a complex national health system is determined by many factors, including variable quality, inadequate financial and human resources, ineffective stewardship, lack of patient empowerment, barriers to continuity of care, inefficient legislative controls and loss of trust between patients and the system [1]. To address these challenges, a high-quality health system must optimize healthcare by consistently providing care that not only achieves but continuously monitors and improves health outcomes, meets the needs of all citizens and is responsive to steadily evolving population needs. Global reports have identified six common dimensions of quality to improve health systems: safety, effectiveness, patient-centredness, timeliness, efficiency and equity [2–4]. To address these dimensions, each country needs to contextually adapt its policies and strategies to meet the needs of its diverse population. To standardize and guide the operationalization of these actions, WHO has developed the National Quality Policy and Strategy (NQPS) initiative, built upon input from experts and diverse country stakeholders. NQPS aims to catalyse systematic performance and improvement while building a culture that supports providers to deliver and users to demand quality care, supported by high-level commitment and a clear governance structure [4]. Given the need to update its existing policies, Malaysia adopted this new international framework to shape its development.

In Malaysia, the Ministry of Health (MOH) launched its Quality Assurance (QA) Programme in 1985, followed by an initial Strategic Plan for Quality in Health in 1998 [5]. This plan provided impetus and direction to institutionalize and optimize healthcare quality. The growth of the health system in the past two decades, including numerous quality and safety initiatives, demanded an updated policy and strategy with a comprehensive implementation plan to monitor the quality of care and health outcomes. Updating the existing plan also aligned with current thinking about the international methodology for national quality policies as defined through the WHO NQPS initiative.

In 2018, the development team requested assistance to engage with the WHO technical experts, culminating in a commitment by the MOH to update and expand its national quality plan. Responding to this need, the Institute for Health Systems Research (IHSR), as the MOH Secretariat for Quality Assurance-Quality Improvement Programme and the WHO Collaborating Centre for Quality Improvement, was designated to spearhead a Technical Working Group (TWG) to develop this new policy, the National Policy for Quality in Healthcare (NPQH). This paper aims to describe the research methodology and process used to develop the NPQH and summarizes its key components.

## Methods

The NPQH technical working group (TWG) conducted a situational analysis using a multi-pronged approach combining historical and new data collection to inform the development of NPQH. Table 1 illustrates the development process timeline, activities and key deliverables.

**Table 1:** Development process timeline, activities and key deliverables

Year	Phase and activities	Key deliverables
2018	Planning and initial phase 1. Proposal to the WHO Office Malaysia to revisit the Strategic Plan for Quality in Healthcare 1998 version 2. Engagement with the WHO Geneva Experts 3. Establishment of the Technical Working Group (TWG)	1. Establishment of Technical Working Group (TWG) 2. Appointment of a WHO technical expert
2019	Development phase (Year 1) 1. Stakeholder mapping 2. Design meeting (with the WHO technical expert) 3. Preparation of a research proposal for situational analysis 4. Sensitization of NPQH development through various channels 5. Conducting situational analysis which adopted three methods: a. Document review (local and international) b. Stakeholders engagement i. General healthcare providers through online survey ii. Public/community through online survey iii. Targeted key stakeholders’ group within and beyond MOH (in-person meeting – with the presence of the WHO technical expert) - Targeted stakeholders (MOH) - Targeted stakeholders (Non-MOH) - Representatives from the Technical/Vertical Programmes (MOH) c. Quality Improvement Initiatives identification and mapping 6. Series of TWG meetings/discussions to conduct SWOT analysis of key findings from the situational analysis to identify key areas of concern 7. Progress update presentations to Quality Committees at the MOH level	1. Identified areas of concern in quality healthcare to inform the development of the new policy and strategy
2020	Development phase (Year 2) 1. Series of TWG meetings/discussions to triangulate findings and identify strategic priorities to address the areas of concern 2. Preparing policy and strategy draft 3. Circulation of policy and strategy draft for review and feedback 4. Refinement of policy and strategy draft	1. Policy and strategy draft
2021	Development phase (Year 3) 1. Series of TWG meetings/discussions to refine policy and action plans 2. Re-engagement sessions with key stakeholders (virtual) a. Targeted stakeholders (MOH) b. Targeted stakeholders (non-MOH) 3. Presentation to top management 4. Further refinement of policy and strategy document 5. Launching of the NPQH	1. Finalized policy and strategy document named as National Policy for Quality in Healthcare
2022	Implementation phase 1. Five-year plan starts 2. Implementation workshops focusing on new activities 3. Meetings to monitor implementation progress	1. Achievement of indicators for each year

### Technical working group (TWG)

The TWG was formed through expansion of the existing QA Technical Committee consisting of representatives from programmes involved with quality initiatives in the MOH. Technical guidance was provided by a WHO-appointed expert throughout the process, focusing primarily on situational analysis and stakeholder engagement, which further enhanced the contributions beyond the MOH participants.

The QA Technical Committee comprises appointed representatives from 12 programmes (Medical, Public Health, Pharmacy, Laboratory, Nursing, Allied Health Science, Food Quality and Safety, Planning, Engineering, Oral Health, Nutrition and Training Management) and key members of the Health Performance Unit. The committee had already been charged with monitoring the national QA Programme through the national indicator approach (NIA), which tracks performance across the MOH healthcare facilities. Each TWG member received an official letter of appointment from the Deputy- Director General (DDG) of Health for Research and Technical Support, indicating support for the initiative from the top level of the MOH.

This TWG was entrusted with the overall planning, design and conduct of the situational analysis, data analysis and facilitation of stakeholder consultative sessions. To accomplish these tasks, the TWG adopted a broad mix of communication methods to coordinate planned activities, including in-person meetings, virtual discussions, official letters and the exchange of emails.

A two-day design meeting involving TWG members with the WHO technical expert was held in January 2019, focusing on three primary objectives:

- To design a plan for a comprehensive situational analysis
- To design a plan of action for stakeholder engagements
- To determine the methodology for obtaining buy-in from stakeholders

Discussions also centred around how to frame questions to obtain the most useful input from stakeholders about their healthcare experiences and how the national health system can best promote quality of care at their respective levels. Following the MOH regulations, the new data collection and stakeholder engagement processes were approved by the Medical Research and Ethics Committee and registered with the National Medical Research Registry (NMRR-19-3522-50030).

### Communication strategy for sensitization of stakeholders

To raise awareness and promote buy-in for the NPQH, the IHSR began communicating about the development of the new policy during all quality-related meetings and others that the IHSR staff attended, including the National QA Committee chaired by the DDG (Research and Technical Support) and the Innovation Steering Committee, which the Secretary-General and the DG of Health co-chair. The IHSR showcased the development of the NPQH in a plenary session during the National QA Convention in October 2019, attended by more than 800 participants from government, private and academic sectors. An information brief [6] was also distributed to attendees, highlighting the rationale for the NPQH and its methodology.

### Guiding elements

**Fig. 1:**
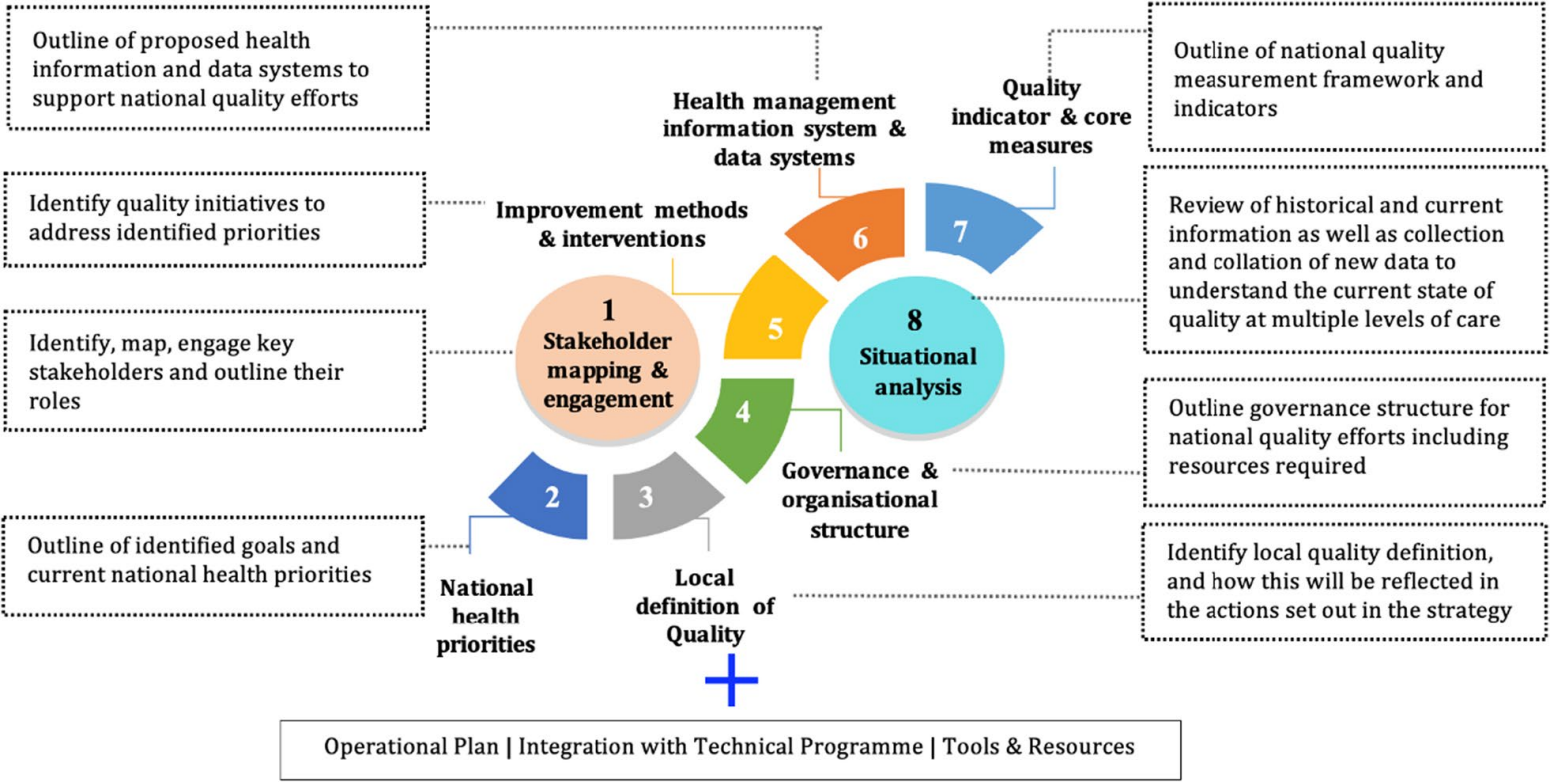
The eight elements applied in developing NPQH guided by the WHO NQPS

The methodology for development of the NPQH was guided by the eight elements described in the WHO NQPS Handbook [4], which was adapted to the Malaysian context (Fig. 1). Two elements were emphasized for developing the policy: (1) stakeholder mapping, including identification of roles and responsibilities, and (2) situational analysis.

### Stakeholder mapping

The TWG members identified potential stakeholders during their first meeting to determine which groups of stakeholders should be engaged. Engagement concentrated on community/patients and stakeholders in healthcare-related ministries involved in delivering healthcare and supporting health services. Existing communication channels, including quality-related meetings and platforms, were identified. Where no existing channels were identified, stakeholders were reached through online surveys. Table 2 outlines the stakeholders’ mapping.

**Table 2:** Stakeholders' mapping, involvement and method of engagement

Stakeholders	Spectrum of involvement [7]	Method of engagement
Community and patients	Consulted and engaged	Online survey
General healthcare workers	Consulted and engaged	Online survey
Healthcare related organizations - Ministry of Health - Ministry of Education - Ministry of Defence - Private sector - Professional Body/Association	Co-design	Series of in-person/virtual engagements

### Situational analysis

Situational analysis was conducted concurrently through three major methods: (i) review of quality-related documents within Malaysia, including regulations, policies and standards; (ii) stakeholder engagement through both in-person meetings and online surveys; and (iii) cataloguing of existing Quality Improvement Initiatives (QII).

#### Document review

A spreadsheet comprising all categories of documents as listed below was developed and distributed to the Head of the Programmes at the MOH level. Each programme identified related documents based on these categories and submitted their data to the research team. The documents included:

i. National health policies and plans and national quality strategies
ii. Quality-related legislations, regulations and statutes
iii. Quality-related government documents (professional training materials, protocols and guidelines)
iv. Performance data
v. Technical and vertical programme reports
vi. Resources to support national quality efforts (domestic budgets, local implementation partners, external agencies and aligned technical programmes)

In addition to the documents submitted by the programmes, the research team also conducted searches within the publication section of the MOH official website. All relevant documents listed were thoroughly reviewed to identify existing regulations and policies to inform the updated NPQH. Furthermore, input was sought from each programme’s focal persons to determine whether specific documents should be considered for inclusion.

The TWG gathered and reviewed a total of 443 documents. They were divided into eight groups to extract data into an Excel template to answer elements in Fig. 1 as below:

- Current national health directions or specific or disease-based programme priorities to guide the NPQH development (Element 2)
- Existing explicit local quality definition or quality domains (Element 3)
- Governance and organizational structure (Element 4)
- Improvement methods and interventions (Element 5)
- Health management information system/data systems (Element 6)
- Set of quality indicators and core measures currently monitored and its performance (Element 7)

Findings from this review were triangulated with other data sources to inform the development of the NPQH. Details of the methods and findings of this document review will be reported in a separate publication.

#### Quality improvement initiatives (QII) mapping/cataloguing

In the Malaysian context, a quality improvement initiative (QII) is defined as “a continuous change process that is data-driven and based on systematically planned action to increase the likelihood of optimal quality of care measured by improved healthcare processes, outcomes and client experience” [8]. Currently, these QIIs address patient safety, effectiveness, people-centeredness, timeliness of service provided, efficiency, accessibility and equity, which are applied throughout the continuum of care from community services through quaternary facilities or life cycles.

A pre-formatted spreadsheet with pre-determined variables was distributed to quality leads in the MOH who oversee QIIs led by the MOH. The specific variables corresponded to Element 4 (governance and organizational structure), Element 5 (improvement methods and interventions), Element 6 (health management information and data system) and Element 7 (quality indicators and core measures). We did not survey initiatives led by other Ministries in the government, in the private sector or those that were specific to health facilities.

The information gathered on each QII was analyzed to understand the extent of implementation and achievements and any identified gaps. They were grouped based on the following categories: (1) leading programme/division; (2) implementation and monitoring level; (3) governance structure; and (4) potential domain of impact on quality: (a) system environment; (b) reducing harm; (c) clinical care improvement; and (d) engagement or empowerment of patient, family or community. Key indicators used in each QII were mapped according to local quality domains. Details of the methods and findings of this QII mapping will be reported in a separate publication.

#### Stakeholder engagement

Stakeholder representatives were divided into three groups for convening: (1) general healthcare providers, (2) public/community, and (3) a targeted group of health leaders from the private, military and education sectors. Engagement of the first two groups was conducted between February and May 2019 through online surveys to obtain opinions from a wide group of healthcare workers and the public who would not be reachable through in-person engagement. The survey links and QR codes were disseminated through various channels, including email, MOH postmasters, Facebook, the official MOH website and WhatsApp groups, among other platforms. The WhatsApp group is used to distribute the survey to family members and the network of the researchers, including quality network at the national and state levels. Table 3 summarizes key stakeholders’ engagement sessions and activities.

**Table 3:** Key stakeholder engagement sessions

Year	Stakeholders involved and numbers	Questions/topic discussed
2019	General healthcare workers (*n* = 587)	1. What is your opinion on quality activities/initiatives at your workplace? 2. What quality activities/initiatives at your workplace can be improved?
	Community and patients (*n* = 800)	1. What matters most during healthcare facility visits? 2. What are the strengths of the healthcare facilities? 3. What suggestions do you have for improvement?
	Targeted key stakeholders (a) Key quality stakeholders in MOH organizations (*n* = 31) - Quality programme leads* - State quality coordinators** - Selected quality champions***	1. What are the quality issues and challenges that you encounter in your programme/state/workplace? 2. How should we define quality in the Malaysian context? 3. How can we move forward to improve the implementation of quality interventions?
	Targeted key stakeholders (b) Key quality stakeholders in non-MOH organizations (*n* = 18) Quality managers or representatives from: - Ministry of Higher Education - Ministry of Defence - Private hospitals - Malaysian Society for Quality in Health (MSQH) - Malaysia Medical Association - Family Medicine Specialist Association	1. What are the quality-related issues that you encounter in your workplace? 2. How can we work together more effectively to improve the quality of health for the nation? 3. How do you receive feedback from patients? 4. What have you done to improve quality that can be replicated in the public sector by MOH?
	Targeted key stakeholders (c) Representatives from MOH Technical Programmes such as HIV/AIDS, maternal health, tobacco and adolescent health (*n* = 13)	1. What are the quality-related issues that you encounter in your programme? 2. How can we foster the integration of quality initiatives into the technical programmes? 3. What are your specific suggestions for strategies to accomplish integration?
2021	Targeted key stakeholders (a) Session with MOH stakeholders (*n* = 62). Representatives from: - Quality leads from MOH programmes or divisions - State quality coordinators - Facility quality champions - Technical programme representatives	1. Proposed local quality and quality improvement definitions 2. Proposed policy and action plan for NPQH 3. Implementation mechanism for NPQH
	Targeted key stakeholders (b) Session with non-MOH stakeholders (*n* = 17) Quality managers or representatives from: - Ministry of Higher Education - Ministry of Defence - Private hospitals - Malaysian Society for Quality in Health (MSQH) - Malaysia Medical Association - Family Medicine Specialist Association	1. Proposed local quality definition 2. Proposed policy and action plan for NPQH

AIDS, acquired immunodeficiency syndrome; HIV, human immunodeficiency virus

^*^MOH’s quality leads are officers who oversee the implementation of various QIIs at the national level

^**^State quality coordinators coordinate quality activities at the state or institution level

^***^Quality champions were identified as the officers who lead/actively involve in a specific quality activity at any level of healthcare and have recognized expertise

##### Online survey among general healthcare providers

General healthcare providers were asked two questions about the implementation of quality improvement initiatives and what can be improved. Convenience sampling was applied. Respondent characteristics were collected, including age, sex, type of health sector and place of work.

##### Online survey among community

An online survey was conveniently distributed to the target sample of Malaysian people aged 18 years old and above with three open-ended questions on the areas that matter most during healthcare facility visits, strengths of the healthcare facilities and suggestions for improvement. Respondent characteristics were collected, including age, sex, estimated income and type of healthcare facility usually used.

Findings for both online surveys were analyzed separately using content analysis to identify major categories of responses for each question. Two TWG members independently reviewed the responses and coded them manually into appropriate main themes and sub-themes of each question asked. After comparing the coding, the teams agreed on the main themes and sub-themes and discussed any areas of disagreement. When consensus could not be reached, a third member was consulted to resolve the matter. Details of the methods and findings of these online surveys will be reported in separate publications.

##### Targeted key stakeholders

Targeted key stakeholder groups were convened twice in 2019 and 2021, respectively. The TWG identified stakeholders based on their roles and expertise in planning, implementing, monitoring and evaluating quality improvement initiatives at their programmes or facilities. The first session was held in-person in July 2019 as part of a 2-week workshop during which all three specific groups were convened separately. Sessions aimed to obtain these groups’ buy-in and seek their input about priorities for quality activities.

The second session was organized virtually in February 2021 to share the NPQH development progress and to obtain feedback on the key content areas of the draft policy and strategies. In both sessions, participants were divided into small groups to discuss a set of predetermined questions. Discussions were summarized and reported back to the entire group. TWG members facilitated and documented these sessions.

The TWG reviewed discussion summaries, meeting notes and groups’ slides presentations to familiarize themselves with the data. Two TWG members independently extracted and coded the data manually following thematic analysis. Results were compared, and any disagreements were discussed with a third member. The relationship between the key findings under each theme was mapped using the Strengths, Weaknesses, Opportunities, Threats (SWOT) framework.

Data from the document review, online surveys and QII mapping were also triangulated in the SWOT analysis to confirm the findings. The preliminary SWOT analysis of the themes underwent many iterations through the inductive process with regrouping before the TWG agreed upon the final version. We noticed the relationship of a few themes: (i) governance for quality and resources; (ii) stakeholder engagement and knowledge exchange, communication and coordination among programmes; and (iii) health management information, quality monitoring and feedback system, and quality indicators and core measures. However, we decided to maintain those as individual areas of concern to be able to see their own SWOT analysis clearly.

### Drafting and refining of the policy

The NPQH first draft was prepared by the IHSR before its circulation to the TWG for comments and input in 2020. The document was organized into four key chapters, shown in Table 4.

**Table 4:** NPQH chapters

Part 1: Introduction	An overview of the national policy and strategy in the global context followed by a description of the Malaysian context and needs. This section highlights the quality journey of the MOH and the existing governance structure for quality and summarizes QII mapping with related achievements
Part 2: Policy and strategy development process	The development process is summarized in graphical and flow chart form to describe the methodology, highlighting the complexity of the health system
Part 3: Policy and strategy	Detailed description of the seven strategic priority areas and associated objectives, plan of action and indicators
Part 4: Mechanism for implementation	Roles and responsibilities of the key players: dissemination, monitoring and evaluation plans for the NPQH

The primary focus of writing was Part 3, in which the policy and strategic plan elements were described, shaped by the NQPS guiding principles and envisioned through the lens of a 5-year time span. This process required many rounds of monthly virtual discussions among the TWG over 1 year to negotiate, deliberate and rationalize each objective, indicator and action plan with consideration of available resources. Feedback from the second engagement sessions in 2021 identified areas of improvement among others, and included refinements of the local quality definition, action plans and indicators were incorporated in the subsequent draft.

The Strategies section was displayed with a clear operational plan covering short, medium and long-term actions, divided into yearly targeted plans, with clearly defined roles at national, state and facility levels and indicators to track progress and achievement.

Part 4 of the document focuses on the mechanism for implementing and monitoring the NPQH. It involves three major components: (i) clearly defined roles and responsibilities of MOH leadership and managers, leaders of QII, leaders and managers at healthcare facilities level, healthcare providers and citizens/clients; (ii) wide distribution of the new policy; and (iii) monitoring and evaluation of the new policy. The plan delineates the roles and responsibilities of the key groups for each area of the plan. Access to the policy will be ensured through various means, including official hard copy distribution and online sources. Close monitoring of implementation will occur through regular meetings of the existing QA Committee with additional members where appropriate. Terms of Reference of existing committees at different levels will be amended to specifically include monitoring of the NPQH achievements and the implementation of the action plan.

### Senior leadership approval

Approval by senior leadership for the NPQH was required at several key points in the development process, including permission to develop the plan. Once underway, input and approvals were obtained for preliminary findings, proposed content and the strategies for communicating with stakeholders. Periodic progress updates were presented to the National QA Committee. The final presentation took place at the Director-General Special Meeting in early April 2021 to obtain input and approval from the top leadership at both the ministry and state levels. External experts and internal reviewers then reviewed the final draft policy before being officially released and launched by the Director-General of Health in October 2021 during the National Seminar for Quality in Healthcare.

## Results

### Key findings

The SWOT analysis uncovered 10 broad areas of concern, each highlighting strengths to be optimized, weaknesses to be prioritized for improvement, opportunities to be pursued and threats to be considered (refer to Additional file 1: Appendix 1). A series of brainstorming sessions among the TWG were organized to explore ideas, addressing each separately. After careful consideration, it was decided that the interrelated themes could be condensed since similar strategies would be used to address them. The resulting seven primary focus areas were considered for developing prioritized objectives, sequences of actions, annual targets and monitoring indicators. Table 5 summarizes the 10 areas of concern with a brief explanation of each and their associated strategic priority areas and objectives.

**Table 5:** The 10 identified areas of concern with formulated strategic priority and objectives

Areas of concern	SP formulated to address the areas of concern	
	SP	Objectives
1. People-centered holistic approach	SP 1: Improving integrated people-centred services	1. Strengthening commitment to improving people-centred care 2. Empowering and engaging people
2. Governance and organizational structure for quality	SP 2: Strengthening governance for quality	1. Strengthening leadership commitment to quality through the monitoring of current organizations’ performances 2. Emphasizing the importance of quality in the MOH at top-level management 3. Strengthening the governance of the Quality Committee/Department/Section 4. Improving resources for Quality
3. Resources		
4. Quality culture	SP 3: Strengthening internalization of quality culture among all healthcare staff	1. Understand the current level of the organization’s quality culture, readiness for change and performances 2. Emphasize employee wellness and welfare 3. Develop, implement and strengthen an engagement plan between top management and healthcare providers 4. Strengthen the reward, incentive and recognition system mechanism 5. Review and optimize the system for healthcare facility accreditation to meet quality of care objectives
5. Stakeholder engagement	SP 4: Enhancing communication and engagement of stakeholders for quality	1. Strengthen the interaction among programmes within the MOH 2. Strengthen the interaction among MOH programmes with other Ministries, private sectors and the community 3. Foster knowledge sharing and knowledge translation platforms in quality improvement activities
6. Knowledge exchange, communication and coordination among programs		
7. Workforce competency and capability towards quality management	SP 5: Building effective capacity and capability for quality	1. Strengthen in-service quality improvement training encompassing technical and soft skills 2. Assessment of the training provided
8. Health management information, quality monitoring and feedback system	SP 6: Enhancing measurement and quality improvement initiatives	1. Reviewing and strengthening the measurement and indicator framework 2. Improving data quality 3. Managing data and linking data sources – strengthening MyHDW 4. Using data for decision-making
9. Quality indicators and core measures		
10. Quality improvement initiatives monitoring and evaluation	SP 7: Strengthening monitoring and evaluation of quality programmes or initiatives	1. Organizing/conducting QII evaluations 2. Dissemination and communication of evaluation results to close the loop

SP- strategic priority; MyHDW- Malaysian Health Data Warehouse

### Other key findings: Local definition of quality

The definition of quality is an overarching concept that informs the entire NPQH. During the document review, existing definitions were sought. The statement on high-quality health systems in the MOH mission statement and two internationally accepted definitions were identified, one from the Institute of Medicine (IOM) [2] and one from WHO [4]. These were used to guide the development of a local definition. Following interactive sessions with policy-makers, document reviews and surveys, the definition of quality was drafted and revised following the second stakeholders meeting. These views have informed the adoption of the following multi-dimensional definition of healthcare quality in Malaysia’s context, which defines high-quality healthcare as safe, timely, effective, equitable, efficient, people-centred and accessible (STEEEPA); innovative; and responsive to the needs of the people, and delivered as a team in a caring and professional manner to improve health outcomes and client experience.

### Other key findings: Quality improvement initiatives (QII)

A total of 40 QIIs were grouped into three primary broad categories: (i) regulatory, (ii) domain-specific QIIs and (iii) QI method. Domain-specific QII categories were further divided into subgroups depending on the domain of their potential impact on quality: (i) system environment, (ii) reducing harm, (iii) clinical care and (iv) engagement and empowerment of patient, family and community [3, 4]. Diagrams to illustrate the QII grouping with associated governance structure are included in the NPQH [8].

The QII mapping provided insights that informed strategies of NPQH. General observations included:

1. Each QII led by a MOH unit has a distinct role in improving the quality of healthcare and has shown significant achievements in the last 5 years (2016–2020)
2. Each QII is supervised by its own committee or sub-committee at the ministry level.
3. Quality Committees at the state, hospitals and health district office levels oversee the implementation of QII at their respective level and report to the specific QII coordinators at the higher level.
4. Each QII has their own set of indicators to track performance with a structured mechanism for data collection and data management.

The key challenge related to QIIs to be addressed by the NPQH was the lack of coordination and interaction among the QII development/monitoring partners within the MOH.

## Discussion

The path of developing NPQH in Malaysia was shaped by several contextual factors related to the governmental structure and the history of its healthcare quality programme. These levers, in turn, led to decisions about the development methods and the processes implemented to create the document.

The new NPQH for Malaysia was built upon an existing Quality Assurance Programme and various quality initiatives, including those implemented at the national level, initiatives involving technical programmes such as maternal and child health and initiatives at the state or facility levels. Existing quality committees in various programmes/divisions at the ministry level were engaged to support this development in ensuring that NPQH was recognized and was inclusive of all key groups of stakeholders.

One critical decision that shaped the creation of NPQH was whether to develop a standalone document or to integrate it into the broader health sector plans. Malaysia decided to recognize the importance of a separate quality policy. This approach allows for greater depth and detail, demonstrates a higher level of visibility for healthcare quality, and allows the use of specific formal planning, implementation and evaluation processes with their own time frames [4]. Despite being a standalone policy, NPQH is firmly aligned with international and national Malaysian health planning frameworks, including the Sustainable Development Goals (SDG) [9] and Universal Health Coverage (UHC) [10], the Malaysia Shared Prosperity Vision 2030 [11], the 12th Malaysia Plan, the Vision and Mission of the Ministry of Health [12] and the MOH Strategic Plan (2021–2025). The NPQH additionally plays a central role in support of the MOH Strategic Plan (2021–2025) under the specific objective of “strengthening healthcare service delivery which is of high quality, sustainable, equitable and affordable”.

While other countries have focused on developing a national quality strategy [13–19, 28], NPQH for Malaysia is both a quality policy and strategy, similar to the approaches taken by South Africa [20], Zimbabwe [21] and Malawi [22]. This document provides an explicit statement of intention and becomes the agreed-upon strategic roadmap that describes how the policy will be implemented and how it may be refined over time. The “quality strategy” provides a link to accelerate the achievement of health goals and priorities, using quality management principles that incorporate quality planning, control and improvement [23, 24]. The NPQH aims to systematically plan for enhanced quality of healthcare by providing an official, explicit policy statement and direction regarding the approach and actions required at all levels of health service delivery across the health system that guide the translation of the policy into practice and the implementation of Malaysia’s 7 Strategic Priority (SP) Areas.

The WHO NQPS’s eight elements served as the leading guide for NPQH development, emphasizing identification of stakeholders and situational analysis to provide a current picture of the state-of-the-art healthcare quality. Namibia and Bangladesh, too, used this framework in developing their new country quality policy and strategy documents [25–27]. We deliberately included implementation as part of a three-pronged approach that includes policy and strategy so that implementation experience informs policy and strategy decisions. By including this real-world perspective from the point of service delivery, implementers have more confidence in the relevance of NPQH and feel more ownership of it [4].

Our methodology for conducting situational analysis was similar to that used in other countries [13–15, 21, 25–29] based on a mixed-methods investigation, which primarily involved document review and engagement of multiple stakeholders followed by mapping of findings using a SWOT analysis. We also used this process to identify current priorities and plans. However, identifying and cataloguing QIIs into different categories provided a unique element that added the value of current QI implementation in practice to the situational analysis. Cataloguing of QII is a crucial step that allows for a holistic view of the quality improvement landscape and enables a more efficient and effective coordination of these initiatives. It identifies redundancies and gaps, enhances resource allocations, opportunities for collaboration and synergy between initiatives and more effective communications and allows for international quality benchmarking.

We intend to continue meaningful interaction with stakeholders across the health system, including the private sector, faith-based groups and cross-sectoral organizations such as professional associations, local governments and academic institutions to ensure that NPQH remains rooted in the present as it plans for the future. This engagement is a critical element and strength of the Malaysia methodology, reflected through the early involvement of key stakeholders within and outside the MOH, including the public. Their active participation and constructive feedback during the engagement sessions aided the development and refinement of the policy.

Another strength of the development process for NPQH, especially given the complexity of the Malaysia Ministry of Health, was the composition of the TWG, which included members of the Quality Assurance Technical Committee. Their substantial participation greatly supported the process by sharing their programmatic quality activities, obtaining relevant quality documents and strategic information, assisting in distributing surveys and facilitating communication and implementation with their leadership and stakeholders at state and facility levels. Given the large number of divisions that implement discrete QIIs, not all could be formally represented in the TWG, although all were actively engaged throughout the development of NPQH.

As with other countries [13–17, 21, 22, 25–28, 30, 31], Malaysia has included governance and leadership for quality as a primary area of focus. While an individual governance structure for each QII provides an advantage for faster growth of these initiatives, the parallel processes have resulted in silos and weaker coordination of initiatives at the national level. Reshaping the existing governance for quality or identification of an organization to lead a quality improvement agenda for the whole country across health sectors is necessary to drive change. Revisiting and strengthening the roles, functions and responsibilities of existing committees, units and individuals may be another option if the existing governance needs to be maintained.

Also similar to other countries, Malaysia has emphasized improving people or client-centered services, highlighting it through the assessment of client satisfaction and experience and by increasing patient engagement and empowerment to drive demand for high-quality health services [14, 15, 17, 21, 22, 25–30, 32]. Robust communication systems to disseminate knowledge among all stakeholders, including learning from best practices within and beyond the country, stand out as a key strategy to ensure that the national quality system will be an active learning system that enables continuous improvement of healthcare quality [33]. NPQH envisages achieving this through establishing a quality hub to serve as a common sharing platform to enhance stakeholder engagement, foster collaboration and promote continuous improvement.

Another common feature of national quality programs is emphasizing capacity building among healthcare staff to improve care continuously [15, 17, 19, 20, 25–28, 31]. Strategies to build healthcare worker ownership of quality are included through investments to develop technical and communication skills for each member of the healthcare workforce and strengthen pre-service, in-service and continuing medical education programs to emphasize QI and patient safety skills. One specific objective is to establish a competency framework for quality to guide the development of technical and communication skills of healthcare professionals across levels of care.

The NPQH aims for an overarching measurement framework and a set of key indicators to track performance based on the local quality definition domains to aid policy-makers to track progress, making informed decisions and drive improvement. Underlying all of these components are the elements of data systems to measure quality and a robust communication system. An important objective is to improve data systems for effective reporting and feedback mechanisms [13, 14, 17, 20, 22, 25–28] to promote a culture of leveraging data for improvement and decision-making.

### Development process challenges and limitations

The major organizational challenge in developing NPQH within the complex Malaysian health sector was that each programme area has its own priorities, which might not easily be captured through broad processes for setting goals, indicators and targets. Disease- or population-specific initiatives may not emerge as priority areas when a national set of objectives is determined. To address this challenge within the TWG, negotiation and compromise were paramount and fostered by an iterative, consensus-building approach. TWG members were able to work through ideas, interpret data, consider different perspectives and commit to a common purpose.

Another challenge was ensuring that TWG members remained committed over three years of development phase despite their primary responsibilities and the turnover of representatives due to transfers and retirements. TWG members’ contributions were recognized through appointment and appreciation letters endorsed by the Deputy Director General to keep them motivated. Achievement of milestones in the Gantt Chart was celebrated to keep the team moving forward. The logistics of scheduling TWG meetings were often challenging, often resulting in the absence of key members. To address this problem, the Secretariat managed communications and corresponded with members via email to complete necessary tasks to ensure that their programme area was represented. To maintain steady progress and motivation, the TWG used an internal WhatsApp group to communicate and stay on track. Dedicated staff for this purpose was a critical enabler to keep processes moving forward.

Engaging stakeholders, particularly those in the private sector, was challenging. More engagement with the private sector associations should be organized in the implementation phase to address this. Additionally, online surveys to engage with the public and healthcare workers may limit in-depth exploration of quality-related issues. To overcome this potential limitation, in-person interviews or focus group discussions should be considered for mid-term strategic plan review.

Although thematic analysis may be influenced by researcher subjectivity, we mitigated this by using two independent coders, resolving disagreements through discussions and conducting peer debriefing. Triangulation with survey and document review data also validated the findings. Additionally, field visits to healthcare facilities for national stakeholders to interact directly with implementers and communities, fostering mutual learning and real-time evidence gathering to guide quality-related discussions and decision-making, should be considered to expand input and validate survey findings.

The coronavirus disease 2019 (COVID-19) pandemic presented negative and positive consequences that affected the development process. Initial timelines were delayed because of urgent COVID-related priorities and secondments. TWG meetings were convened virtually, and the second major engagement session was also convened virtually in 2021. Although in-person communications were sacrificed, the virtual platform offered an opportunity for more participants to join, especially those from distant states who did not need to travel to participate.

### Anticipated implementation challenges

Implementation of NPQH across the health sector poses several challenges, especially in facilities not overseen by the MOH, namely the private and education sectors. Since the NPQH is an overarching policy document, it is not enforced and is essentially voluntary for these sectors to follow. Continued and ongoing engagement will be an important strategy for the NPQH implementation. Another possible strategy to engage these sectors is through collaboration with the Malaysian Society for Quality in Health, a non-profit national accreditation organization that can integrate the NPQH indicators and related action plans into the accreditation standards.

We also expect that monitoring and evaluating this new policy will pose challenges, requiring a new systematic approach to producing reports, establishing a communication system for disseminating information and promoting transparency to stakeholders. This process will be necessary for continued engagement, ensuring that data are translated into action and for accountability of the MOH. We plan to involve the TWG in monitoring implementation, establishing a database for indicator achievement, convening routinely scheduled meetings and conducting workshops focused on initiating the new activities agreed upon in the strategic plan. Existing platforms for broader engagement with healthcare workers across sectors through the National QA Convention will be optimized.

### Future research

Findings from the SWOT analysis provide several opportunities for future research exploration in areas of concern. For example, a holistic organizational culture assessment is needed to enhance understanding and influence local healthcare culture for quality and safety improvement. Another crucial future research area pertains to workforce competency in quality healthcare, which can inform the development of capacity-building programmes for current and future healthcare quality positions. On top of that, research on the impact of the NPQH on healthcare quality and participation of the healthcare providers in integrating the national policy and strategy into their clinical practices is crucial for understanding how policy changes affect the delivery of healthcare services to inform future policy development and implementation strategies.

## Conclusion

The multi-methods approach used in the process of situational analysis to develop the NPQH has enriched the formulation of a more comprehensive policy and strategy to address the quality of healthcare in Malaysia. Quantitative and qualitative findings that were extracted and mapped using SWOT analysis enabled an insightful understanding of the current state of quality in healthcare in Malaysia. Engaging the right group of stakeholders from the beginning of the development phase, seeking their input through various methods, has increased buy-in for developing this new policy.

The NPQH has identified and fostered a process of broad buy-in across the health sector. It includes a comprehensive range of domains, which aim to accelerate improvement, focusing on people-centred priorities and key stakeholder concerns, including the positive attitudes of healthcare staff, availability and use of data from quality initiatives, a new overarching governance structure for quality and a formal process for monitoring and evaluation of quality programmes and initiatives. Aligned with national goals for health in Malaysia, the NPQH is an important framework to strengthen the national approach to quality and offers promise to improve the health and life of all Malaysians.

### Supplementary Information

**Additional file 1.** Appendix 1: SWOT Analysis.

## Data Availability

The dataset that supports the findings of this article is not publicly available. Requests for data can be obtained from the corresponding author on reasonable request and with permission from the Director General of Health, Malaysia.
