# Supplementary material for: Development of the National Policy for Quality in Healthcare for Malaysia
Source: Health Res Policy Syst. 2023 Nov 14;21:119. doi: 10.1186/s12961-023-01063-w (PMC10644427; doi:10.1186/s12961-023-01063-w)
Supplement: Supplementary file 1 — Additional file 1. Appendix 1: SWOT Analysis. [file 12961_2023_1063_MOESM1_ESM.docx]

Appendix 1: The SWOT Analysis

| **1. Governance and Organisational Structure for Quality** | |
| --- | --- |
| **Strengths** | **Weaknesses** |
| 1. The existence of one standardised vision for quality, which is supported by capable leaders at multiple levels of the organisation 2. Established structures for individual QIIs | 1. Lack of coordination, integration and collaboration among QIIs within and beyond MOH leading to a siloed health system 2. Facility management capability needs to be enhanced to help improve workflow |
| **Opportunities** | **Threats** |
| 1. The availability of an existing platform to develop a national policy for quality based on the WHO’s UHC initiative 2. Potential for restructuring of organisational structure to consolidate quality programs 3. Alignment with the 12^th^ Malaysia Plan (2021) provides opportunities for innovation related to information technology | 1. Inertia-continued segmentation of quality programs and initiatives |

| **2. Knowledge Exchange, Communication & Coordination Amongst Stakeholders** | |
| --- | --- |
| **Strengths** | **Weaknesses** |
| 1. Good networking amongst the relevant ministries enables the sharing of crucial information (National Security Council, *JK Bencana,* etc) 2. Availability of easily accessible and updated information through trusted online sources/ physical platforms 3. Multiple platforms for sharing knowledge, especially in the government sector, facilitate public awareness of health issues | 1. Ineffective communication stemming from inadequate understanding of relevant knowledge and ineffective communication skills 2. Perceived lack of confidence in the data quality (questionable data quality & unverified data) may decrease effectiveness at communicating evidence 3. Undue bureaucracy impedes the sharing of information leading to a lack of participation from beyond MOH |
| **Opportunities** | **Threats** |
| 1. Optimising existing website/ social media etc 2. Exploration of best practices with the private/ other sectors (especially about cost management) 3. Partnership with NGOs, private etc. 4. Review of current policies related to information sharing 5. Availability of information technology advancements provides opportunities for innovations and integration of QII | 1. Misinterpretation by the public as well as misuse of the information by outsiders 2. High expectations from the public 3. Gaps between knowledge & practice in both the government & non-government sectors 4. Inadequate participation from agencies beyond MOH |

| **3. People-Centred Holistic Approach** | |
| --- | --- |
| **Strengths** | **Weaknesses** |
| 1. A few programmes utilising the people-centred holistic approach have been adopted by the community and healthcare facilities, e.g. KOSPEN, *Hospital Mesra Ibadah*, Mother-Friendly Hospital, Father Friendly Hospital 2. Improving people-centred care through value-adding service projects such as medication collection by “drive through”, post, or self-collection through pigeons hole, have been implemented | 1. The perceived lack of leadership in driving and emphasising people-centred care resulting in a fragmented people-centred holistic “culture” 2. Lack of cooperation among staff of various categories as people-centred care is considered optional by the majority of people 3. The subjective measurement of people-centred care makes its assessment and evaluation difficult 4. Lack of specific resource allocation to develop or enhance the methods in gathering data to measure the performance of people-centred care |
| **Opportunities** | **Threats** |
| 1. Quality domain has already been included as an element of Universal health coverage (UHC) 2. Some NGOs are interested in improving patient-centred care services 3. Patient and family engagement is already highlighted as an essential part of people-centred care | 1. Lack of awareness of the importance of people-centred care among policymakers and healthcare staff |

| **4. Health Management Information & Quality Monitoring and Feedback System** | |
| --- | --- |
| **Strengths** | **Weaknesses** |
| 1. Health Management Information Systems (HMIS) is already operational 2. MyHDW is MOH’s main data warehouse with multiple data collection systems feeding in data to cater to different needs and purposes | 1. Lack of an integrated data system resulting in an uncoordinated data collecting system and issues related to data quality 2. Lack of routine data on community input to drive health system planning 3. Ongoing manual data collection with poor feedback mechanisms 4. Absence of disaggregated data to determine whether disparities in healthcare equity exists 5. Data quality problem |
| **Opportunities** | **Threats** |
| 1. Data centralisation utilising the MyHDW platform as well as making individual patient-level data available for analysis 2. The government policy on Digital Government for data-driven decision making | 1. Data security regarding public data sharing of the organisation’s quality performance may lead to threats to the provider’s reputation |

| **5. Resources** | |
| --- | --- |
| **Strengths** | **Weaknesses** |
| 1. Generally, facilities are conveniently located, clean, and well-arranged with dedicated and passionate staff 2. NGO providing medical assistance (especially dialysis fees & services) | 1. Certain facilities face the challenges of inadequate infrastructure, such as equipment and parking lots 2. Insufficient human resources dedicated to quality and high turnover rate due to the absence of a clear future pathway for quality professionals 3. Challenge in developing a succession plan for future champions/leadership in quality |
| **Opportunities** | **Threats** |
| 1. Resource sharing with the private sector as an alternative to distributing the burden 2. NGOs providing medical assistance (especially dialysis fees & services) to assist public hospitals in accommodating to large patient volumes | 1. Insufficient funding for QI initiatives and activities, including training |

| **6. Workforce Capacity & Capability for Quality Improvement** | |
| --- | --- |
| **Strengths** | **Weaknesses** |
| 1. Several quality-related training modules are available, which are acknowledged and awarded points through the Continuing Professional Development Programme (CPD) 2. Some elements under QI are incorporated in the induction course for new entrants (e.g. patient safety) 3. Availability of some champions from various methodologies | 1. Sub-optimal workforce technical competency, soft skills and communication 2. Lack of adequate utilisation of quality trained staff in positions to achieve impact 3. Inadequate utilisation of local quality training modules as they are not regularly improvised to be comparable internationally 4. Awareness & knowledge about quality during undergraduate training is limited 5. Insufficient coordination to make quality improvement training and capacity building more accessible, leading to a suboptimal number of quality champions 6. Lack of provision of periodical incentives 7. The workforce is already overburdened, and the perception that quality improvement is a separate entity from core duties |
| **Opportunities** | **Threats** |
| 1. Ongoing training has produced a pool of passionate and dedicated QI practitioners and pensioners whose skills can be tapped 2. Presence of self-learning platforms as well as opportunities to learn from other sectors | 1. Budget constraints 2. Negative perception towards staff who are placed in quality as less capable, resulting in a lack of recognition of these staff |

| **7. Quality Indicators & Core Measures** | |
| --- | --- |
| **Strengths** | **Weaknesses** |
| 1. Indicators are monitored using readily available data 2. Good commitment from the ground in terms of data provision 3. Some mechanisms to verify data are already in place (i.e. audit KPI) | 1. Oversupply and duplication of indicators due to parallel reporting systems lead to redundancy and overburdening of facilities 2. Indicators are less guided by current national health priorities and lack comparability with international standards. 3. Inadequate action in identifying factors contributing to SIQ as well as post-evaluation monitoring 4. Data from the private sector/ universities/ MOD is not routinely captured, resulting in a less holistic representation of quality 5. No centralisation in governing various indicators from multiple initiatives leads to a lack of coordination |
| **Opportunities** | **Threats** |
| 1. To revisit, realign and harmonise existing indicators towards Malaysia's plan goals and the Ministry of Health’s Strategic Plan 2. To create collaboration with the private sector/ universities/ MOD (i.e. use statisticians to analyse our data) | 1. Poor quality inaccurate data may hamper decision-making at the national level 2. Political influence may result in focus directed towards specific achievable issues only |

| **8. Stakeholder Engagement for Quality** | |
| --- | --- |
| **Strengths** | **Weaknesses** |
| 1. Stakeholders, including the private sector, have shown support to improve integration and coordination of quality programs in healthcare 2. Open and transparent communication between stakeholders improves access to decision-making processes, resulting in more efficient and responsive services 3. The contribution of opinions and insights by key stakeholders have been incredibly valuable in the early stages of the planning and development processes | 1. Lack of stakeholder engagement activities resulting in limited opportunities for their input and concern about policies 2. Inadequate involvement from private sectors 3. Inappropriate stakeholders are identified |
| **Opportunities** | **Threats** |
| 1. Private healthcare facilities possess strong quality systems which can be tapped through collaborative partnerships 2. It brings people together to pool knowledge, experience, and expertise to co-create solutions and share best practices | 1. Stakeholders may develop a lack of confidence in the project team, either as a result of feeling their concerns and opinions have not been addressed or that risks are not being adequately managed |

| **9. Quality Improvement Initiatives M&E** | |
| --- | --- |
| **Strengths** | **Weaknesses** |
| 1. Existing successful quality activities include a wide range of improvement innovations. 2. Availability of quality experts from various disciplines 3. Existence of quality-sharing platforms to share successful best practices | 1. Inadequate internal and external evaluations of the impact of quality improvement initiatives 2. Sub-optimal engagement at various implementation levels, including the community 3. The culture of siloed working has resulted in a lack of communication among programmes/ organisations |
| **Opportunities** | **Threats** |
| 1. Optimize the use of digital information technology to improve M&E 2. Platforms of communication are already available in abundance to publicise to relevant quality stakeholders | 1. Time-consuming 2. Over-stretched and overstressed human resources for quality due to insufficient staff |

| **10. Quality Culture** | |
| --- | --- |
| **Strengths** | **Weaknesses** |
| 1. MOH Corporate Culture incorporates quality culture 2. Existence of an established platform for QA/QI training and sharing 3. The implementation of some quality initiatives at facility/ district/ state levels is included as part of the performance measurement | 1. Corporate culture is not well internalised or embraced 2. Persistence of a punitive or blaming culture would dampen the practice of learning from errors 3. Overstretched insufficient staff with a lack of recognition for quality eventually hinders the practice of quality culture |
| **Opportunities** | **Threats** |
| 1. Try different methods in assessing and managing local healthcare cultures 2. Nurture and reinforce positive deeper values through early professional education curriculum 3. Macro-policy environment can be utilised to encourage a shared way of thinking | 1. Resistance from staff due to possible unresolved issues of insufficient human resource |
